# PD-L1检测方法在非小细胞肺癌的研究进展

**DOI:** 10.3779/j.issn.1009-3419.2019.01.08

**Published:** 2019-01-20

**Authors:** 雪晶 郭, 赫 曹, 建娅 周, 建英 周

**Affiliations:** 310000 杭州，浙江大学医学院附属第一医院呼吸内科 Department of Respiratory Disease, Thoracic Disease Center, The First Affiliated Hospital, College of Medicine, Zhejiang University, Hangzhou 310000, China

**Keywords:** PD-L1, 非小细胞肺癌, 免疫组织化学技术, 酶联免疫吸附测定, PD-L1, Non-small cell lung cancer, Immunohistochemistry, Enzyme-linked immunosorbent assay

## Abstract

PD-1/PD-L1抑制剂在非小细胞肺癌（non-small cell lung cancer, NSCLC）一线和二线治疗中均占重要地位，为NSCLC治疗提供了新的策略。已有的临床试验表明有效检测PD-L1的表达是免疫抑制剂治疗的关键环节。然而，目前尚缺乏PD-L1检测的金标准。近几年国内外免疫组织化学技术（immunohistochemistry, IHC）和酶联免疫吸附测定（enzyme-linked immunosorbent assay, ELISA）不断创新，因而在PD-L1检测中有良好的应用前景。文章就PD-L1检测方法在NSCLC中的研究进展进行了总结和展望。

## Abstract (en)

PD-1/PD-L1 inhibitors play an important role in the first-line and second-line treatment of non-small cell lung cancer (NSCLC), indicating a new treatment strategy of NSCLC. Completed clinical trials have shown that effective detection of PD-L1 expression is the key to the use of immunosuppressive agents. However, the gold standard for PD-L1 detection has still lacked. In recent years, immunohistochemistry (IHC) and enzyme-linked immunosorbent assay (ELISA) have been continuously innovated, which accounts for good prospect in PD-L1 detection. The research progress of PD-L1 detection methods in NSCLC is summarized in this review.

目前肺癌在世界范围内呈现发病率高，且肿瘤相关死亡率居于第一的特点^[1]^。非小细胞肺癌（non-small cell lung cancer, NSCLC）是最常见的组织学类型，约占肺癌病例的85%^[2]^。对于早期和一些局部晚期的NSCLC，手术是最佳的选择，但是许多患者难以避免术后的复发^[3]^。以铂类为基础的化疗是晚期NSCLC的标准治疗，但对生存期的改善有限。过去几年里，以针对特定基因突变为主的分子靶向治疗取得了重大进展，却仍避免不了因获得性耐药而导致后续的肿瘤恶化^[4]^。

近年来，随着肿瘤生物学与免疫学的交叉渗透，以逆转免疫抑制和重塑肿瘤微环境为主的免疫治疗蓬勃发展。值得注意的是，在KEYNOTE-010等一系列高质量临床试验中，程序性死亡因子-1（programmed death 1, PD-1）/程序性死亡因子配体-1（programmed death ligand 1, PD-L1）抑制剂在NSCLC中展现了持久的抗肿瘤效应^[5-7]^。抗PD-1和抗PD-L1单抗价格昂贵且有免疫相关副反应，找准潜在的获益人群是关键。国际上有报道的生物标志物包括PD-L1、肿瘤突变负荷、肿瘤浸润的淋巴细胞等，而目前有比较明确临床试验依据的是PD-L1的表达^[5, 7]^。近来关于PD-L1作为生物标志物的检测方法取得一定进展，本文对此作一综述。

## 1 PD-L1检测概述

PD-L1是PD-1最主要的配体，属B7家族成员，主要以膜性形式（mPD-L1）广泛存在于肿瘤细胞和免疫相关细胞，部分以可溶性形式（sPD-L1）存在于血液和胸腔积液^[8]^。PD-1/PD-L1信号通路能诱导与维持外周T细胞的耐受，是肿瘤免疫逃避的关键分子机制，导致肿瘤发生发展^[9]^。PD-L1检测方法包括免疫组织化学技术（immunohistochemistry, IHC）、酶联免疫吸附测定（enzyme-linked immunosorbent assay, ELISA）、定量免疫荧光（immunofluorescence, IF）和流式细胞术（flow cytometry, FC）等多种，其中IHC以其高效快速的特点在mPD-L1检测应用最广泛，而ELISA作为稳定便捷的检测方法在sPD-L1占据主要地位。

## 2 IHC

### 2.1 标本来源

目前在免疫治疗相关的临床试验中只采用组织学标本进行PD-L1的定量检测。

最近，多项研究证实了细胞学标本用于PD-L1定量检测的可行性和有效性。Heymann等^[10]^的一项评估细胞学、小活检与手术切除NSCLC标本的研究显示，通过检测取自188例患者的214个标本中PD-L1的表达量，细胞学和组织学（包括手术切除和组织小活检）标本的PD-L1阳性率没有差异（*P*=0.083），其中23个有多种标本的病人中PD-L1表达一致者占21个（91%）。Skov等^[11]^的研究中进一步发现，在肺组织的同一部位取材获得86个成对的细胞学蜡块和组织学标本高度相关（*R*^2^=0.87-0.89）。此外，Sakakibara等^[12]^研究显示了通过超声内镜引导下的经支气管针吸活检（endobronchial ultrasound transbronchial needle aspiration, EBUS-TBNA）获得的细胞学标本兼有高度一致性和微创的优点。综合以上研究结果发现，在临床实践中，尤其对于晚期NSCLC患者和组织学标本获取困难的情况下，以EBUS-TBNA为代表的细胞学标本可以作为新的选择。但是在其被纳入常规临床应用前，需进行严格的大规模验证研究和建立一系列细胞采集的标准化操作流程。

此外，最新的一项研究提出了循环肿瘤细胞（circulating tumor cells, CTCs）作为非侵入性方法可有效检测晚期NSCLC患者的PD-L1表达^[13]^。首先在ISET平台上富集和分析外周血标本的CTCs，再行IHC检测其表达。该研究评估了71个配对的肺癌组织和CTCs样本中PD-L1表达量，两者一致性达93%（CTCs敏感性55%，特异性100%）。这显示了CTCs在实时监测晚期NSCLC患者PD-L1表达上具有较大潜力。

### 2.2 标本处理

蛋白质的稳定性受到多方面的影响，包括缺血时间、固定类型、固定时间、抗原修复技术、存储条件和标本存储时间，故检测前的标本处理至关重要^[14]^。标本缺血需尽可能短，建议在取材后立即浸入固定液中。而10%中性缓冲福尔马林溶液因能较好地保存组织细胞的形态结构和抗原性而成为标准的固定液。活检标本至少固定5 h，手术切除标本固定24 h-72 h，要控制好固定时间，过度固定也会导致组织中蛋白质交联而影响免疫组化抗体试剂的渗透^[15]^。标本存储时间应在3年以内。研究显示新鲜的肿瘤标本和3年以内的存档标本均可用于PD-L1检测^[16]^。这意味着对于部分患者可避免再次活检的风险，达到已获取的肿瘤标本的最大使用。另外，关于细胞学标本的处理标准有待进一步优化验证。Lloyd^[17]^的研究表明Cellient自动化细胞块系统可能是PD-L1检测细胞学样本的首选方法。目前可以接受福尔马林固定石蜡包埋同时含有足够肿瘤细胞（至少50个-100个）的细胞蜡块用于检测^[10]^。

### 2.3 检测抗体

IHC是利用抗原与抗体特异性结合，通过化学反应使标记抗体的显色剂显色来对组织细胞抗原进行定位、定性及相对定量的方法。现在已有22C3、28-8、SP142、SP263已经获得美国食品药品监督管理局（Food and Drug Administration, FDA）批准应用于临床。22C3与28-8对应Dako诊断平台，前者的阳性标准规定≥1%肿瘤细胞染色为阳性，≥50%肿瘤细胞染色为强阳性，后者阳性标准是≥1%肿瘤细胞染色。而SP142与SP263对应Ventana诊断平台，前者阳性标准是≥10%肿瘤和（或）肿瘤浸润性免疫细胞染色，后者是≥25%肿瘤细胞染色。此外，还存在实验室检测抗体，如E1L3N。

近几年，国际上对于不同抗体检测的一致性研究已有不少报道。美国学者对90例NSCLC手术切除标本分别用22C3、28-8、SP142和E1L3N检测肿瘤细胞和免疫细胞的PD-L1表达情况，由13位病理学家评估染色百分比。结果显示，采用SP142抗体在肿瘤细胞和免疫细胞中检测到的PD-L1明显减少，不同抗体检测到的肿瘤细胞评分的一致性为0.813（95%CI: 0.815-0.839），而免疫细胞评分的一致性为0.277（95%CI: 0.222 -0.334）^[18]^。2016年德国进行了一项比较22C3、28-8、SP142和SP263在肺腺癌和鳞癌的染色模式研究，在15例手术切除标本的染色中，采用28-8和22C3单抗染色的标本中肿瘤细胞染色比例相似，相比之下，SP142在4例标本中的肿瘤细胞染色较少，SP263在9例标本中显示了更多的肿瘤细胞染色比例^[19]^。2017年同样评估这4种抗体一致性的蓝印计划（The Blueprint Project）开展，39例NSCLC标本的实验结果表明了当使用22C3、28-8和SP263分析时，染色的肿瘤细胞百分比是可比的，而SP142的检测结果显示染色的肿瘤细胞整体较少^[20]^。德国一致性研究和蓝印计划在SP263同22C3、28-8单抗的可比性结论上有所出入，可能是样本量过少引起。同年，Ratcliffe等^[21]^用22C3、28-8和SP263分别检测500个NSCLC的存档标本，设定肿瘤细胞胞膜阳性染色百分比包括1%、10%、25%和50%多个截断值作为PD-L1阳性标准，不同单抗之间的总体符合率均 > 90%。另外，对于以E1L3N为代表的实验室检测抗体的争议仍存在，不同研究阐明的一致性变异大^[22, 23]^，有待日后更多的研究加以改进和论证。综合以上结果可以发现，22C3、28-8和SP263的检测结果具有相似的肿瘤细胞阳性百分比，一致性较高，而SP142染色阳性的肿瘤细胞较少。由此，我们可以推定22C3、28-8和SP263单抗对肿瘤细胞的染色结果具有可交换性，意味着其在未来的临床应用上将有较大潜力。

### 2.4 检测结果判读

IHC的结果判读主要通过人为定性或半定量，有一定的主观性。所以国际上存在共识，需要设立专门的实验室和要求病理学家具备足够的经验。尤其目前不同药物之间截断值不一，病理学家们在日常实践中更应该反复熟悉各种PD-L1截断值的参考图像。基于人工判读的现状，我国学者提出应用计算机辅助图像分析技术进行精确定量的判读方法^[24]^。计算机采集每个切片的5个视野图像，计算染色程度比值[（棕色区域面密度/蓝色区域面密度)×棕色平均光密度]，将空白对照片的比值定义为阈值，与人工判读（阳性强度×阳性细胞百分数）比较109例肺腺癌中PD-L1的阳性结果，发现一致率为88.073%。可以发现，图像分析技术和人工判读的一致性较好，并且前者更具客观性。

## 3 ELISA

sPD-L1保留有PD- 1结合域，故在肿瘤患者体液中具有一定的生物学活性，尽管在其功能和释放机制上仍有争议之处，但是sPD-L1检测也可以作为临床应用的一项选择，尤其对于无法获得足够肿瘤标本的患者。当前，ELISA是最常用的检测方法。近几年，随着对sPD-L1的研究深入及其抗体的制备创新，双抗体夹心ELISA法取得不少进展。2011年我国苏州大学经过反复筛选和比较，建立了以鼠单抗2H11为捕获抗体和以生物素标记的10D7为检测抗体的ELISA检测体系，检测范围为0.195 ng/mL-12.5 ng/mL，*R*² > 0.99，显示其具有良好的特异性和稳定性^[25]^。之后国外研究发现成对小鼠单克隆抗体H1A和B11对人PD-L1细胞外域有特异性作用，采用H1A作为平板固定捕获抗体，用生物素化的B11作为检测抗体^[26]^。而今年日本的一项研究开发了新的ELISA检测系统，PD-1-Ig融合蛋白取代了常规ELISA中的固定化捕获抗体。该研究利用配体受体反应，能够定量检测到具有与PD-1结合能力的功能性sPD-1。在75例NSCLC患者的血浆样本检测中，新的ELISA检测到sPD-L1阳性者29例（38.6%），平均吸光度是（0.292±0.461），而常规ELISA检测到阳性者8例（10.6%），平均吸光度是（0.051±0.027），阐明了新的ELISA灵敏度高于常规ELISA^[27]^。

## 4 其他

近年来IF和FC开始应用于PD-L1检测领域。IF利用抗原抗体特异结合与荧光素标记技术，由于荧光素所发的荧光可在荧光显微镜下检出，人为对抗原进行细胞定位。Velcheti等^[28]^在多种单抗中筛选出了符合标准的5H1抗体进行IF，自动化统计荧光强度，最后以定量IF评分评估NSCLC组织中PD-L1的表达量。而FC是利用流式细胞仪对荧光染色单克隆抗体标记的肿瘤细胞进行多参数、快速的定量分析技术，更具客观性。2017年Caldwell等^[29]^报道了RK-10多肽在多种肿瘤类型中显示出与PD-L1结合的高度特异性和灵敏性，并且对其进行荧光素标记（RK-10-Cy5），用FC检测组织、细胞和血液标本中的PD-L1表达量。现如今，IF和FC针对PD-L1的特异性抗体研发后劲不足，制备工艺尚未成熟，因此在PD-L1检测领域有待进一步创新和推广。

## 5 PD-L1检测的临床价值

### 5.1 指导治疗

分子靶向和免疫抑制在肿瘤治疗领域大放异彩，标志着肿瘤治疗已经进入精准医疗的时代。其中，有效检测生物标志物从而正确选择获益人群是精准治疗的关键环节。大多数研究证据表明，PD-L1是用于免疫治疗选择的可靠预测性生物标志物。在POPLAR研究中，对于铂类化疗后进展的NSCLC，Atezolizumab以12.6个月的总生存期（overall survival, OS）获益超越多西他赛，且OS的延长与增加的PD-L1表达密切相关，PD-L1表达阳性的患者OS为15.5个月，明显高于多西他赛（9.2个月）^[30]^。类似地，Keynote001^[7]^和Keynote 010^[5]^研究也发现对于PD-L1≥1%的NSCLC患者二线治疗选择pembrolizumab显著优于化疗，同时显示PD-L1≥50%的患者更多临床获益，确立根据PD-L1表达指导免疫治疗的重要价值。另一方面，对于PD-L1检测阴性的患者，应改变治疗策略，如联合免疫治疗^[31]^。目前PD-L1作为预测标志物具有两个诊断级别，伴随诊断指相应药物使用必须进行的检测，补充诊断是非必须的检测但可提供治疗相关信息。经过大量的临床研究，现已确立PD-L1检测是Pembrolizumab的伴随诊断，肿瘤细胞表达PD-L1≥50%的患者一线治疗使用Pembrolizumab获益最大，PD-L1≥1%的患者以其作为二线治疗获益最大。

### 5.2 预后判断

临床上，肺癌患者的预后主要依据TMN分期，病理类型和肿瘤标志物等。目前，较多研究发现sPD-L1高表达与较差的临床预后相关。有研究评估NSCLC患者组织B7-H1的表达，结果显示B7-H1与肿瘤体积增大、淋巴结转移和晚期的TMN分期高度相关，并提出B7-H1是不良预后的独立因素^[32]^。Okuma等^[33]^进一步研究证实经ELISA检测血浆高sPD-L1的肺癌患者OS明显缩短（13.0个月*vs* 20.4个月，*P*=0.037）。并且，在接受胸部放疗的患者中，经ELISA检测血浆sPD-L1基线水平较低者相比较高者获得更长的OS获益（27.8个月*vs* 15.5个月，*P*=0.005）^[34]^。而最近一项研究针对肺癌伴恶性胸腔积液的患者，收集积液细胞蜡块标本进行免疫组化染色，发现低免疫细胞PD-L1表达往往预后总生存较差（*P*=0.004）^[35]^。我们可以推断PD-L1的检测有助于预后判断，尤其是以抽取外周血为主的sPD-L1检测方便易行，有望成为临床上判断肺癌预后的有力工具。

## 6 小结

综上所述，在肺癌免疫研究飞速发展的今天，PD-L1的检测方法不断创新。IHC是当今检测方法主流，用以筛选免疫治疗的获益人群。虽然目前检测抗体和检测平台不一，但各种抗体的一致性检验带给我们信心，22C3、28-8和SP263单抗的检测结果具有可交换性，我们可以预见未来有广阔的临床应用前景。另外，针对sPD-L1检测的ELISA是后起之秀，可简便易行地判断临床预后，但在未来仍需要更多关于PD-L1作为NSCLC预后标志物的研究。
